# Revealing microbial recognition by specific antibodies

**DOI:** 10.1186/s12866-015-0456-y

**Published:** 2015-07-02

**Authors:** Áurea Simón-Soro, Giuseppe D’Auria, M. Carmen Collado, Mária Džunková, Shauna Culshaw, Alex Mira

**Affiliations:** Department of Health and Genomics, FISABIO Foundation, Center for Advanced Research in Public Health, Avda. Cataluña 21, 46020 Valencia, Spain; The Institute of Agrochemistry and Food Technology, Spanish National Research Council (IATA-CSIC), 46980 Valencia, Spain; Infection and Immunity Research Group, Glasgow Dental School, School of Medicine, College of Medical, Veterinary and Life Sciences, University of Glasgow, Scotland, UK

**Keywords:** Immunoglobulin, Flow cytometry, Pyrosequencing, 16S rRNA, Opsonization, Human microbiome

## Abstract

**Background:**

Recognition of microorganisms by antibodies is a vital component of the human immune response. However, there is currently very limited understanding of immune recognition of 50 % of the human microbiome which is made up of as yet un-culturable bacteria. We have combined the use of flow cytometry and pyrosequencing to describe the microbial composition of human samples, and its interaction with the immune system.

**Results:**

We show the power of the technique in human faecal, saliva, oral biofilm and breast milk samples, labeled with fluorescent anti-IgG or anti-IgA antibodies. Using Fluorescence-Activated Cell Sorting (FACS), bacterial cells were separated depending on whether they are coated with IgA or IgG antibodies. Each bacterial population was PCR-amplified and pyrosequenced, characterizing the microorganisms which evade the immune system and those which were recognized by each immunoglobulin.

**Conclusions:**

The application of the technique to healthy and diseased individuals may unravel the contribution of the immune response to microbial infections and polymicrobial diseases.

**Electronic supplementary material:**

The online version of this article (doi:10.1186/s12866-015-0456-y) contains supplementary material, which is available to authorized users.

## Background

There are extensive data describing the human microbiome, and germ free animal models demonstrate its intricate relationship with the host [1]. However, less is understood about this relationship in humans, either in health or disease. The coating of microorganisms by antibodies may promote defense against infection, and regulate the immune response to the microbiota to limit potentially damaging responses, thus maintaining homeostasis in human-associated microbial communities [1, 2]. The recognition of microbes by different immunoglobulins (Igs) plays a vital role in the host-microbiome relationship [3], but the associations between Ig classes and specific groups of bacteria and fungi remain relatively poorly characterized [4]. Flow cytometry allows the separation of bacterial cells according to their population structure [5, 6] and to the fluorescence emitted by antibodies specifically bound to different human Igs [7, 8]. These labeled populations of bacteria can be characterized by second-generation sequencing of PCR-amplified microbial rDNA genes, to provide a description of the bacterial and fungal diversity and taxonomic composition [9].

**Fig. 1:**
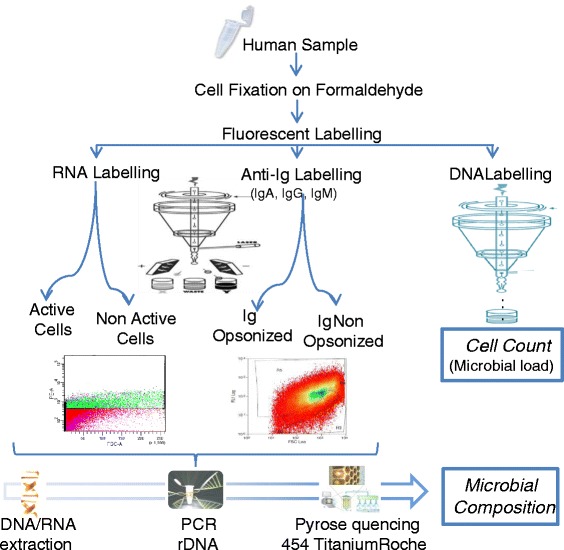
A mixed Flow Cytometry-Next Generation Sequencing strategy to identify human host-microbial associations. Body samples (saliva, faeces, urine, mucosa, milk, etc.) are disaggregated by vortexing and mild sonication. Microbial cells are fixed in 4 % paraformaldehyde previous to staining with fluorescent markers to detect cells (e.g. by DNA markers [6]), active cells (e.g. by RNA markers [17]) and specific antibodies (e.g. by anti-human IgA) through a flow cytometer. Microbial load can also be accurately estimated by cell counting. Cells are sorted depending on whether they are opsonized with either IgA or IgG antibodies. Each bacterial population can then be PCR-amplified and pyrosequenced, characterizing the microorganisms which evade the immune system and those which are recognized by each immunoglobulin. The application of the technique to healthy and diseased individuals may unravel the contribution of the immune response to microbial infections and polymicrobial diseases

We have applied a combination of flow cytometry cell sorting coupled with pyrosequencing to identify the bacteria that are coated with specific antibodies, avoiding the sample bias imposed by bacterial culture (Fig. 1). We show the potential of this mixed approach by estimating the proportion of Ig-coated bacteria in saliva, faecal, oral biofilm and breast milk samples, as well as identifying the specific bacterial genera that are either coated and uncoated by different Igs.

## Methods

### Sample collection and processing

All donors signed informed consent and the sampling protocol was approved by the Dirección General de Salud Pública (DGSP) ethical committee (Valencian Health Authority, Spain) for the faecal and oral samples; and the Bioethics Subcommittee of Consejo Superior de Investigaciones Científicas (CSIC) for the breast milk samples. For the oral samples, unstimulated saliva samples and supragingival dental plaque from 5 individuals that had never suffered from dental caries and 4 individuals with active caries were collected in sterile tubes 24 h after tooth brushing. Six faecal samples were stored in RNALater within 2 h from sampling and stored at −20 until fixation in formaldehyde. Healthy volunteer mothers with term deliveries were given written instructions for standardised collection of breast milk samples. Before sample collection, the breast was cleaned with an iodine swab to reduce bacteria residing on the skin, and breast milk was collected manually and after discarding first drops collected into a sterile milk collection unit, then immediately frozen and stored at −20 °C until analysis. A total of 12 breast milk samples corresponding to colostrum and mature milk were collected. All samples were centrifuged at 7500 g for 7 min to collect microbial cells and washed twice in physiological solution (NaCl 0.9 %). Cells were immediately fixed in 4 % paraformaldehyde overnight at 4 °C. Fixed cells were washed twice and stored at −20 °C in 50 % ethanol until use. On the experiment day, samples were washed in sterile saline solution and disaggregated 20 s in a sonicator bath, model Raypa VCI-50 at low ultrasound intensity.

### Flow cytometry

Samples were suspended in sterile saline solution with 5 % albumin to prevent non-specific antibody binding, then stained with (i) anti-human IgA or IgG labelled with FITC (Invitrogen catalog # A18782 and A18806); and (ii) the DNA-binding fluorophor SYTO62 (Invitrogen catalog # S11344) according to the manufacturer instructions. Anti-mouse IgA or IgG labeled with FITC (Invitrogen catalog # M31101 and A24525) were used for isotype controls.

Cell sorting was performed with the MoFloTM XDP flow cytometer (Beckman Coulter Inc.) using Argon 488 nm (blue) laser (200 mW power) and the 635 nm (red) diode laser (25 mW power) as light sources. The lasers were aligned using Flow-CheckTM (10 μm) and Flow-SetTM (3 μm) fluorospheres (Beckman Coulter, Inc.). Emission filters were 520/30 for FITC and 680/30 for SYTO62 respectively. Proper fluorescent labeling was assessed by fluorescence and confocal microscopy (Additional file 1: Figure S1). Cells were separated according to their fluorescence in both the FITC and SYTO62 channels (Ig-coated bacteria) or the SYTO62 channel only (non-coated bacteria).

### Taxonomic identification

DNA from the Ig-coated and non-coated fractions with more than 5000 cells was extracted using the MasterPure™ Complete DNA and RNA Purification Kit (Epicentre Biotechnologies), following the manufacturer’s instructions, with the addition of a lysozyme treatment [10]. The 16S rRNA gene was amplified using universal bacterial primers 8 F and 533R with sample-specific barcodes, as previously described [10]. Purified PCR products were mixed in equimolar amounts and sequenced using the 454 GS-FLX pyrosequencer (Titanium chemistry, Roche). The resulting 16S rRNA reads were end-trimmed in 10 bp sliding windows with average quality value > 20, then length (200 pp) and quality filtered (average Q > 20). Taxonomic assignments were performed with the RDP classifier [11], and to estimate total diversity, sequences were clustered at 97 % nucleotide identity over 90 % sequence alignment length to obtain rarefaction curves. Principal Coordinates Analysis (PCoA) was performed with FastUnifrac [12], comparing the 16S-estimated composition with a phylogenetic approach that takes into account both taxonomically assigned and unassigned reads.

## Results and discussion

Formaldehyde-fixed samples were stained with (i) anti-human IgA or IgG labelled with FITC; and (ii) the DNA-binding fluorophor SYTO62. Proper fluorescent marking was confirmed by fluorescence and confocal laser scanning microscopy (Additional file 1: Figure S1). Anti-mouse IgA or IgG, of identical isotype to the anti-human antibodies, labeled with FITC, (Invitrogen) were used to control for non-specific binding. Cells were separated in the flow cytometer according to their fluorescence in both the FL1-FITC and FL8-SYTO62 channels (Ig-coated bacteria) or the SYTO62 channel only (non-coated bacteria). DNA from the Ig-coated and non-coated fractions was extracted and the 16S rDNA gene amplified and pyrosequenced, in order to describe bacterial composition of the sorted populations. An outline of the experimental pipeline is described in Fig. 1. The density and number of cells that could be sorted from each fraction varied among samples (Additional file 2: Table S1). Although PCR and subsequent pyrosequencing was achieved with as few as 5000 sorted bacteria, a larger number of cells is recommended for accurate description of microbial composition [13].

**Fig. 2:**
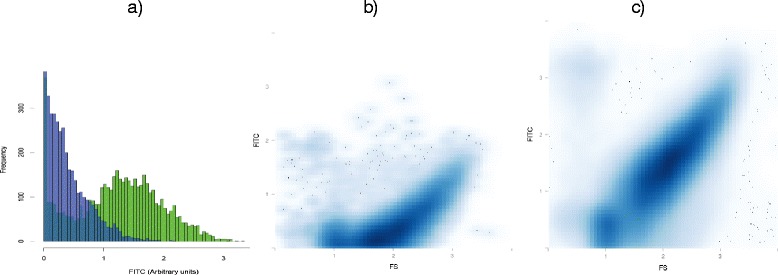
Fluorescence activated cell sorting of opsonized bacteria in a faecal sample stained with anti-IgA markers. The frequency histogram (**a**) represents the events displaying FITC fluorescence with anti-human IgA (green) and anti-mouse IgA (blue) markers. The latter is used as isotype control (non-specific binding). Thus, the green area to the right corresponds to IgA-coated micro-organisms. The green area overlapping with non-specific binding indicates a lack of fluorescence, corresponding to non-opsonized cells. FITC-based histograms are typically bimodal, with the two peaks corresponding to Ig-coated and non-coated populations. The corresponding scatterplots for anti-mouse and anti-human IgA labeling are shown in (**b**) and (**c**), respectively. The region above anti-mouse IgA binding was gated to select true opsonization. FS = Forward scatter, which correlates with cell size

**Fig. 3:**
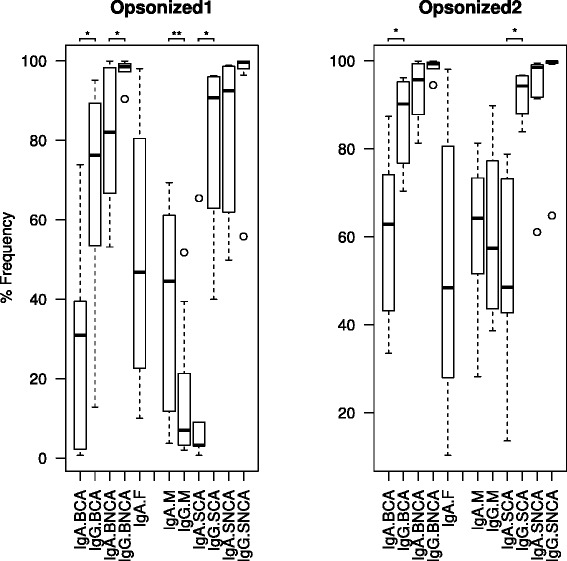
Ig-coating levels in different human samples. Boxplots show the mean values and variation in IgA- and IgG-opsonization levels for oral biofilm (B), milk (M), fecal samples (F), and saliva (S). Asterisks indicate statistically significant differences between IgA and IgG coating (Wilcoxon test, *p* < 0.05). For saliva and oral biofilm, samples from Caries-free (NCA) and Caries-bearing (CA) individuals are available. Data are shown for a conservative (Opsonized 1) and a non-conservative (Opsonized 2), upper estimate of Ig-coating. Individual data are shown in Additional file 2: Table S1

When a frequency histogram was built depending on the anti-Ig marker fluorescence, a bi-modal distribution was typically observed, with a small peak at low fluorescence and a larger peak at high fluorescence intensities, for both IgA and IgG (Fig. 2). This suggests that only a low proportion of bacteria are apparently ignored by, or able to evade the humoral immune system. Specifically, the average proportion of IgA-opsonized bacteria in the subjects analyzed in the current proof of concept study ranged from 52.3 % for faecal samples, 73.6 % for saliva, 78.4 % for the oral biofilm, and 63.7 % for breast milk (Fig. 3, Additional file 2: Table S1). An even larger proportion of bacteria were coated with IgG; 93.8 % for saliva, and 92.1 % for the oral biofilm. This could potentially reflect a biological difference in the IgG/IgA antibodies specificity in oral samples, but different efficiencies in anti-human Ig antibodies binding or in fluorescence emission cannot be ruled out. In previous studies, 45 % of faecal bacteria were found to be opsonized by IgA in samples from healthy donors, a proportion that increased to 69 % under inflammatory episodes [14]. However, given that the number of secreted IgA was estimated to be 10^7^ times higher than the number of gut microbes [15], higher proportions can potentially be achieved. The greater proportion of opsonized bacteria detected in the oral samples analyzed in the current study is intriguing and could reflect local immune regulation requirements or an antibody mediated defense to limit microorganisms binding the gastrointestinal tract. It is also interesting to note a significantly higher Ig-coating level in saliva and oral biofilm samples from healthy individuals compared to caries-bearing patients (Fig. 3). This indicates that the immune activity in caries-free individuals could be more competent than in patients with compromised oral health.

**Figure 4:**
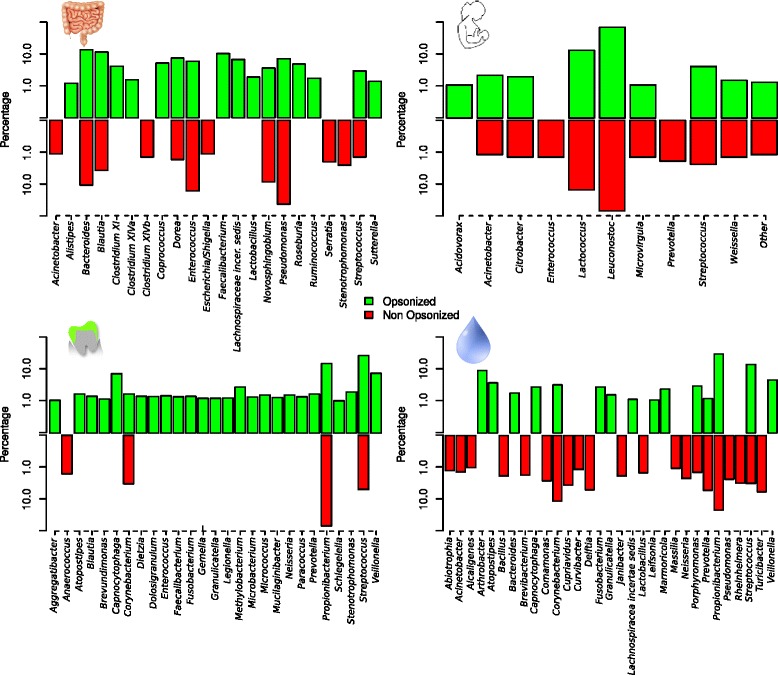
Bacterial composition of IgA-coated and non-coated populations. Graphs show the proportion of bacterial genera within the opsonized and non-opsonized populations in individual samples from feces, breast milk, oral biofilm (dental plaque) and saliva as estimated by 16S rDNA pyrosequencing of fluorescence-activated sorted cells. The four samples are from different individuals. Only bacteria found at a frequency >1 % are shown. Some bacterial genera appear at similar proportions in both the Ig-coated and non-coated populations. Others appear only within the opsonized fraction (strong IgA-specificity) whereas some microorganisms are present only within the non-opsonized fraction (immune evasion or non-recognition)

Pyrosequencing of Ig-marked and unmarked cells identified which bacteria were opsonized and non-opsonized by specific antibodies. In both saliva, faeces, oral biofilm and breast milk samples, the frequency of many bacterial genera in the two sorted fractions was different, suggesting regional differences and a particular affinity of the antibody for some microorganisms (Fig. 4). As a consequence, specific bacteria appear to be able to evade opsonization, including *Escherichia* and *Stenotrophomonas* in the gut, or *Enterococcus* and *Prevotella* in breast milk. Others, like *Veillonella* and *Fusobacterium*, appear always opsonized both in saliva and dental plaque. In the oral biofilm samples, most bacterial genera appeared to be IgA-coated.

**Figure 5:**
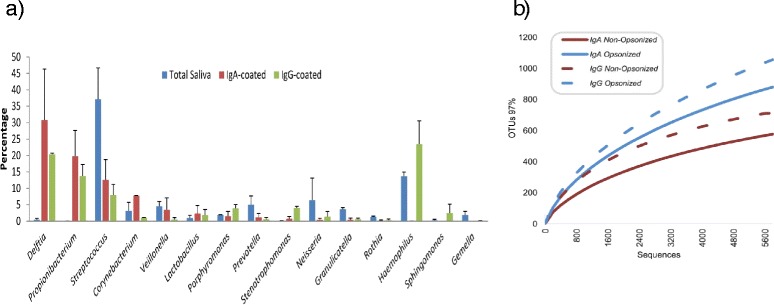
Diversity of Ig-coated and uncoated bacteria in human saliva. Saliva samples collected 24 h after toothbrushing (*n* = 16) were stained with fluorescent markers for bacterial DNA, IgA and IgG, and sorted in three groups: IgA-coated bacteria, IgG-coated bacteria and uncoated, non-opsonized bacteria. **a** Bacterial composition at the genus level for total saliva samples, as well as for the IgA- and IgG-coated fractions. The bacterial composition appeared to be different between the different fractions. **b** Rarefaction curves relating pyrosequencing effort to the estimated number of species (OTUs at 97 % sequence identity). The non-opsonized fractions display a lower diversity and different taxonomic composition to opsonized populations

The sum of the proportion of IgA- and IgG coated bacteria for the same samples was in most cases higher than 100 %, indicating that a large proportion of cells were coated by both immunoglobulins, as suggested by other studies [16, 17]. However, the bacterial composition in the IgA- and IgG-coated fractions was different (Fig. 5). As shown by a Principal Coordinates Analysis in saliva samples, the composition of the IgA- and IgG-opsonized fractions did not cluster together. In addition, the opsonized and non-opsonized populations occupied different positions in the PCoA space, indicating that the taxonomic groups coated by antibodies are different from those that are ignored or undetected by them. For instance, the genera *Delftia* or *Propionibacterium* were found at low frequencies in the saliva samples, but were highly opsonized by both IgA and IgG (Fig. 5a). *Streptococcus*, on the other hand, was very frequent in the same saliva samples, but moderately opsonized. Differential opsinization was found for *Haemophilus*, which was found to be coated by IgG but not IgA (Fig. 5a). Data for saliva samples from individual CA021 show that the most common genera in the IgA-opsonized fraction were *Propionibacterium*, *Streptococcus*, *Arthrobacter*, *Veillonella* and *Atopostipes*, whereas the latter three were absent in the non-opsonized fraction (Fig. 4). In the future, the sequencing of IgA-, IgG- and IgM-coated microbes in larger numbers of samples should confirm whether there is Ig-specific opsonization.

Finally, rarefaction curves of species-level bacterial diversity show that the opsonized population is more diverse than the non-opsonized one (Fig. 5b). In future studies, we anticipate that the sequencing of the non-opsonized fractions will reveal those micro-organisms that are undetected or ignored by specific antibodies. Although the current work was done with titanium chemistry FLX pyrosequencing and sequences were under 500 bp long on average, current advances in this and other technologies are expected to allow read lengths over 900 bp shortly, allowing taxonomic assignment at the species level. This will no doubt be necessary for accurate description of antibody-microbial specificity, as current read lengths are mainly reliable at the genus level [18].

Another aspect that can readily be observed in flow cytometry scatter plots in environmental samples is the presence of aggregated populations as evidence by their larger size and specific shapes [5]. Our own observations in human samples through fluorescence and confocal microscopy revealed that some of those large-size clusters are bacterial aggregates and others are formed by bacteria bound to host cells like detached buccal epithelial cells. These aggregates can by sorted and subsequently identified by 16S rDNA pyrosequencing (Additional file 3: Figure S2). In individual CA060, for instance, 70 % of a bacterial aggregate in a saliva sample was found to be formed by *Porphyromonas*, *Streptococcus*, *Prevotella*, *Propionibacterium*, *Veillonella*, and unidentified *Bacteroidetes*. This approach paves the way to unravel the nature of bacterial aggregation in body fluids with important repercussion for active and passive immunization approaches and novel antimicrobial strategies. For instance, aggregated microorganisms may be less accessible to antibodies and partially escape opsonization.

The mixed FACS-pyrosequencing approach presented here can also be applied to identify fungi, by using fungal-specific fluorescent markers and subsequent sequencing of PCR-amplified fungal ITS or 28S rRNA regions [19]. In addition, an RNA-binding fluorophor like pyronin can be used to quantify, separate and sequence-identify active bacteria [6, 17]. In our saliva samples (n = 6), 31-43 % of bacteria appeared to be marked by pyronin, suggesting that a large portion of organisms in the oral cavity can be transient or inactive (Additional file 1: Figure S1). In the future, marking of IgA and IgG with different fluorescent markers could be used in the same sample, in order to distinguish individual cells coated by both of these antibodies. Finally, micro-organisms cell counts can be used to accurately calculate bacterial and fungal load, which can be related to the body fluid chemical and biological components. That way, features of the immune response can be associated to microbial composition and density, providing insights about functioning of the immune system and suggesting potential biomarkers of health and disease conditions.

## Conclusions

The approach presented here involves the identification of Ig-detected and ignored microbes in healthy and diseased individuals [14, 20]. This approach offers novel insights into understanding host-microbe homeostasis in health and its disruption in myriad diseases, ranging from oral diseases (e.g. dental caries or periodontal disease) to gut disorders (e.g. Crohn’s disease, ulcerative colitis, or irritable bowel disease [20]) and even Ig-recognition of tumor cells. Considering immune recognition and opsonization in healthy individuals as a reference, deviations from that balanced microbe-immune interaction can potentially be related to microbial-mediated disorders, and the characterization of individual-specific opsonization profiles may also prove fruitful in diagnostic and therapeutic strategies for personalized medicine.

## Additional files

Additional file 1: Figure S1. Staining of microbial cells for Fluorescence Activated Cell Sorting. Confocal laser microscopy images of saliva samples collected 1 h after toothbrushing from individual CATV01 stained with different fluorescent markers. (a) Saliva sample stained with the fluorophors SYTO62 (DNA labeling, red fluorescence) and FITC (anti-human IgM, green fluorescence). Image corresponds to the transversal section Z10, using a 63 × magnification and a 1.5 zoom. (b) Saliva stained with the RNA-binding fluorophor Pyronin-Y (RNA labelling, red fluorescence). The intensity of fluorescence can be related to RNA production and therefore to cell activity and both active and non-active cells are observed. The image is a projection of all transversal sections in the sample, performed at 63x magnification with a 3.0 zoom in a Leica HCX PLAPO confocal microscope. In both cases, stained and unstained bacteria can be separated by fluorescence detection cell sorting in order to describe the corresponding microbial populations by rRNA PCR and pyrosequencing. Additional file 2: Table S1. Estimated number of Ig-coated cells as detected by Fluorescence-Activated Cell Sorting in saliva, oral biofilm, faeces and breast milk human samples. Additional file 3: Figure S2. Identification of bacterial composition in aggregates. The scatterplot shows microbial cells in a saliva sample according to their size (X axis) and the IgG coating, as indicated by their FITC-fluorescence using anti-human IgG markers (Y axis). The large-size aggregate (indicated with a black circle) was separated by fluorescence-activated cell-sorting and its DNA pyrosequenced after PCR of the 16S rRNA gene, describing its bacterial diversity at the genus taxonomic level. The bacterial populations displaying FITC fluorescence values below 10 correspond to non-opsonized cells.
