# Supplementary material for: Revealing microbial recognition by specific antibodies
Source: BMC Microbiol. 2015 Jul 2;15:132. doi: 10.1186/s12866-015-0456-y (PMC4489363; doi:10.1186/s12866-015-0456-y)
Supplement: Additional file 1: Figure S1. — Staining of microbial cells for Fluorescence Activated Cell Sorting. Confocal laser microscopy images of saliva samples collected 1 h after toothbrushing from individual CATV01 stained with different fluorescent markers. (a) Saliva sample stained with the fluorophors SYTO62 (DNA labeling, red fluorescence) and FITC (anti-human IgM, green fluorescence). Image corresponds to the transversal section Z10, using a 63 × magnification and a 1.5 zoom. (b) Saliva stained with the RNA-binding fluorophor Pyronin-Y (RNA labelling, red fluorescence). The intensity of fluorescence can be related to RNA production and therefore to cell activity and both active and non-active cells are observed. The image is a projection of all transversal sections in the sample, performed at 63x magnification with a 3.0 zoom in a Leica HCX PLAPO confocal microscope. In both cases, stained and unstained bacteria can be separated by fluorescence detection cell sorting in order to describe the corresponding microbial populations by rRNA PCR and pyrosequencing. [file 12866_2015_456_MOESM1_ESM.pdf]

Supplementary Figure 1. Staining of microbial cells for Fluorescence Activated Cell Sorting.

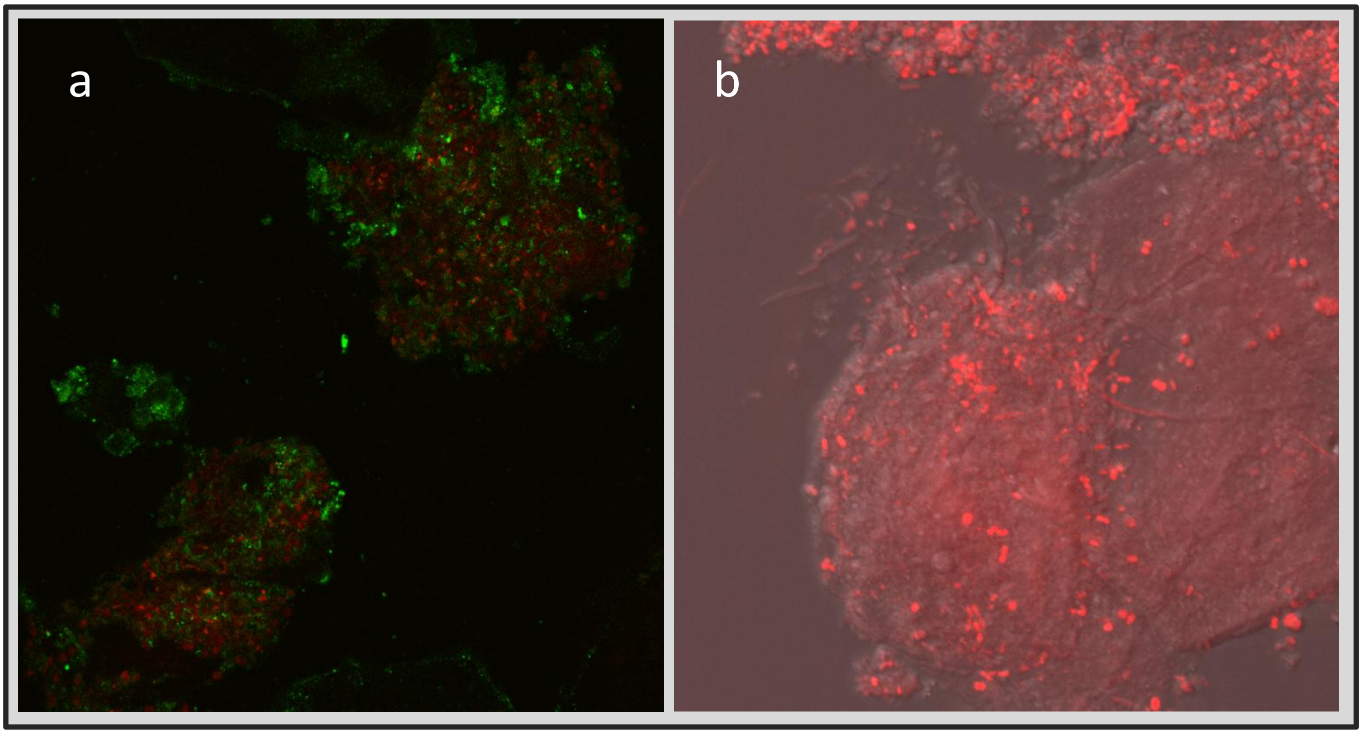

Confocal microscopy images of saliva samples collected 1 h after toothbrushing from individual CATV01 stained with different fluorescent markers. (**a**) Saliva sample stained with the fluorophors SYTO62 (DNA labeling, red fluorescence) and FITC (anti-human IgM, green fluorescence). Image corresponds to the transversal section Z10, using a 63x magnification and a 1.5 zoom. (**b**) Saliva stained with the RNA-binding fluorophor Pyronin-Y (RNA labelling, red fluorescence). The intensity of fluorescence can be related to RNA production and therefore to cell activity and both active and non-active cells are observed. The image is a projection of all transversal sections in the sample, performed at 63x magnification with a 3.0 zoom in a Leica HCX PLAPO confocal microscope. In both cases, stained and unstained bacteria can be separated by fluorescence detection cell sorting in order to describe the corresponding microbial populations by rRNA PCR and pyrosequencing.
