# Supplementary material for: Revealing microbial recognition by specific antibodies
Source: BMC Microbiol. 2015 Jul 2;15:132. doi: 10.1186/s12866-015-0456-y (PMC4489363; doi:10.1186/s12866-015-0456-y)
Supplement: Additional file 2: Table S1. — Estimated number of Ig-coated cells as detected by Fluorescence-Activated Cell Sorting in saliva, oral biofilm, faeces and breast milk human samples. [file 12866_2015_456_MOESM2_ESM.pdf]

Supplementary Table 1. Estimated number of Ig-coated cells as detected by Fluorescence-Activated Cell Sorting in saliva, oral biofilm, breast milk and fecal human samples.

(*) indicates the number of events marked by the DNA-labelling fluorophore SYTO62; its number is larger than the addition of the following two columns, as it includes the events marked with anti-mouse Ig (potential non-specific binding). (1) Estimated Ig-coated cells calculated as a proportion out of the addition of opsonized and non-opsonized sorted events. (2) Upper estimate of opsonization, calculated as a proportion of total events, including potential non-specific binding (1-non-stained/total cells).

	Ig type	Total number of events (*)	Events not stained with anti-Ig marker	Events stained with anti-Ig marker	Estimated % opsonization (1)	Estimated % opsonization (2)
Saliva Samples						
NCA.01	IgA	277519	24000	24827	50.85	91.35
NCA.01	IgG	317523	313	292643	99.89	99.90
NCA.03	IgA	2329	907	902	49.86	61.06
NCA.03	IgG	3379	1189	1501	55.80	64.81
NCA.13	IgA	64277	331	21999	98.52	99.49
NCA.13	IgG	52681	90	23514	99.62	99.83
NCA.12	IgA	52489	794	9689	92.43	98.49
NCA.12	IgG	39581	300	8024	96.40	99.24
NCA14	IgA	7818	79	5939	98.69	98.99
NCA.14	IgG	5736	19	5042	99.62	99.67
NCA.15	IgA	35682	2826	7651	73.03	92.08
NCA.15	IgG	8144	8	7012	99.89	99.90
NCA.16	IgA	46420	391	34086	98.87	99.16
NCA.16	IgG	56675	48	55120	99.91	99.92
CA.54	IgA	230726	199300	1407	0.70	13.62
CA.54	IgG	121442	19563	13018	39.96	83.89
CA.57	IgA	13154	3523	351	9.06	73.22
CA.57	IgG	7648	605	3649	85.78	92.09
CA.58	IgA	4201	891	1686	65.42	78.79
CA.58	IgG	3812	134	3011	95.74	96.48
CA.59	IgA	713765	367303	12357	3.25	48.54
CA.59	IgG	29276	966	24839	96.26	96.70
CA.60	IgA	1497908	858524	26979	3.05	42.69
CA.21	IgA	16225	1175	1938	62.25	92.76
CA.21	IgG	9756	933	3235	77.61	90.44
Oral Biofilm (dental plaque)						
NCA.01	IgA	18184	2372	3711	61.01	86.96
NCA.01	IgG	22496	411	14326	97.21	98.17
NCA.03	IgA	92386	17260	19580	53.15	81.32
NCA.03	IgG	21918	1213	11427	90.40	94.47
NCA.13	IgA	6341	720	1880	72.31	88.65
NCA.13	IgG	18137	121	8910	98.66	99.33
NCA.12	IgA	68915	2946	13444	82.03	95.73
NCA.12	IgG	54898	390	22898	98.33	99.29
NCA14	IgA	651156	7032	220752	96.91	98.92

NCA.14	IgG	617821	276	383777	99.93	99.96
NCA.15	IgA	755467	1056	355902	99.70	99.86
NCA.15	IgG	1061756	3168	445649	99.29	99.70
NCA.16	IgA	250136	89	200327	99.96	99.96
CA.54	IgA	21936	12466	284	2.23	43.17
CA.54	IgG	17576	1605	4383	73.20	90.87
CA.57	IgA	7412	4928	34	0.69	33.51
CA.57	IgG	2885	854	126	12.86	70.40
CA.58	IgA	2387	617	402	39.45	74.15
CA.58	IgG	4254	163	3179	95.12	96.17
CA.59	IgA	125125	15812	44696	73.87	87.36
CA.59	IgG	136475	6532	54707	89.33	95.21
CA.60	IgA	119635	44510	26248	37.10	62.80
CA.60	IgG	96302	22438	25723	53.41	76.70
CA.61	IgA	42324	15679	5141	24.69	62.95
CA.61	IgG	93251	9776	37207	79.19	89.52
Breast milk						
Sample 1	IgA	379082	70943	56319	44.25	81.29
Sample 1	IgG	554106	59580	21512	26.53	89.25
Sample 2	IgA	596418	115046	50852	30.65	80.71
Sample 2	IgG	641861	65202	2424	3.58	89.84
Sample 3	IgA	219940	105690	85690	44.77	51.95
Sample 3	IgG	229950	100236	12563	11.14	56.41
Sample 4	IgA	118347	30491	53294	63.61	74.24
Sample 4	IgG	40248	9176	9853	51.78	77.20
Sample 5	IgA	87003	39300	45063	53.42	54.83
Sample 5	IgG	57503	34097	2657	7.23	40.70
Sample 6	IgA	75132	46264	5964	11.42	38.42
Sample 6	IgG	72164	44279	1212	2.66	38.64
Sample 7	IgA	846301	274013	35373	11.43	67.62
Sample 7	IgG	988582	485195	15098	3.02	50.92
Sample 8	IgA	27776	10903	15418	58.58	60.75
Sample 8	IgG	44849	13534	1000	6.88	69.82
Sample 9	IgA	69826	50126	1930	3.71	28.21
Sample 9	IgG	72476	38785	25250	39.43	46.49
Sample 10	IgA	96112	28192	49888	63.89	70.67
Sample 10	IgG	92235	20951	4014	16.08	77.29
Sample 11	IgA	150886	41487	93945	69.37	72.50
Sample 11	IgG	145377	60451	3190	5.01	58.42
Sample 12	IgA	525752	256581	35622	12.19	51.20
Sample 12	IgG	513255	312540	6512	2.04	39.11
Fecal samples						
1	IgA	647170	580473	64717	10.03	10.31
2	IgA	638335	124016	510668	80.46	80.57
3	IgA	668882	12976	655504	98.06	98.06
4	IgA	653784	470780	137295	22.58	27.99
5	IgA	431783	291352	138171	32.17	32.52
6	IgA	287278	102468	163748	61.51	64.33
