# Supplementary material for: Revealing microbial recognition by specific antibodies
Source: BMC Microbiol. 2015 Jul 2;15:132. doi: 10.1186/s12866-015-0456-y (PMC4489363; doi:10.1186/s12866-015-0456-y)
Supplement: Additional file 3: Figure S2. — Identification of bacterial composition in aggregates. The scatterplot shows microbial cells in a saliva sample according to their size (X axis) and the IgG coating, as indicated by their FITC-fluorescence using anti-human IgG markers (Y axis). The large-size aggregate (indicated with a black circle) was separated by fluorescence-activated cell-sorting and its DNA pyrosequenced after PCR of the 16S rRNA gene, describing its bacterial diversity at the genus taxonomic level. The bacterial populations displaying FITC fluorescence values below 10 correspond to non-opsonized cells. [file 12866_2015_456_MOESM3_ESM.pdf]

Figure S2. Identification of bacterial composition in aggregates.

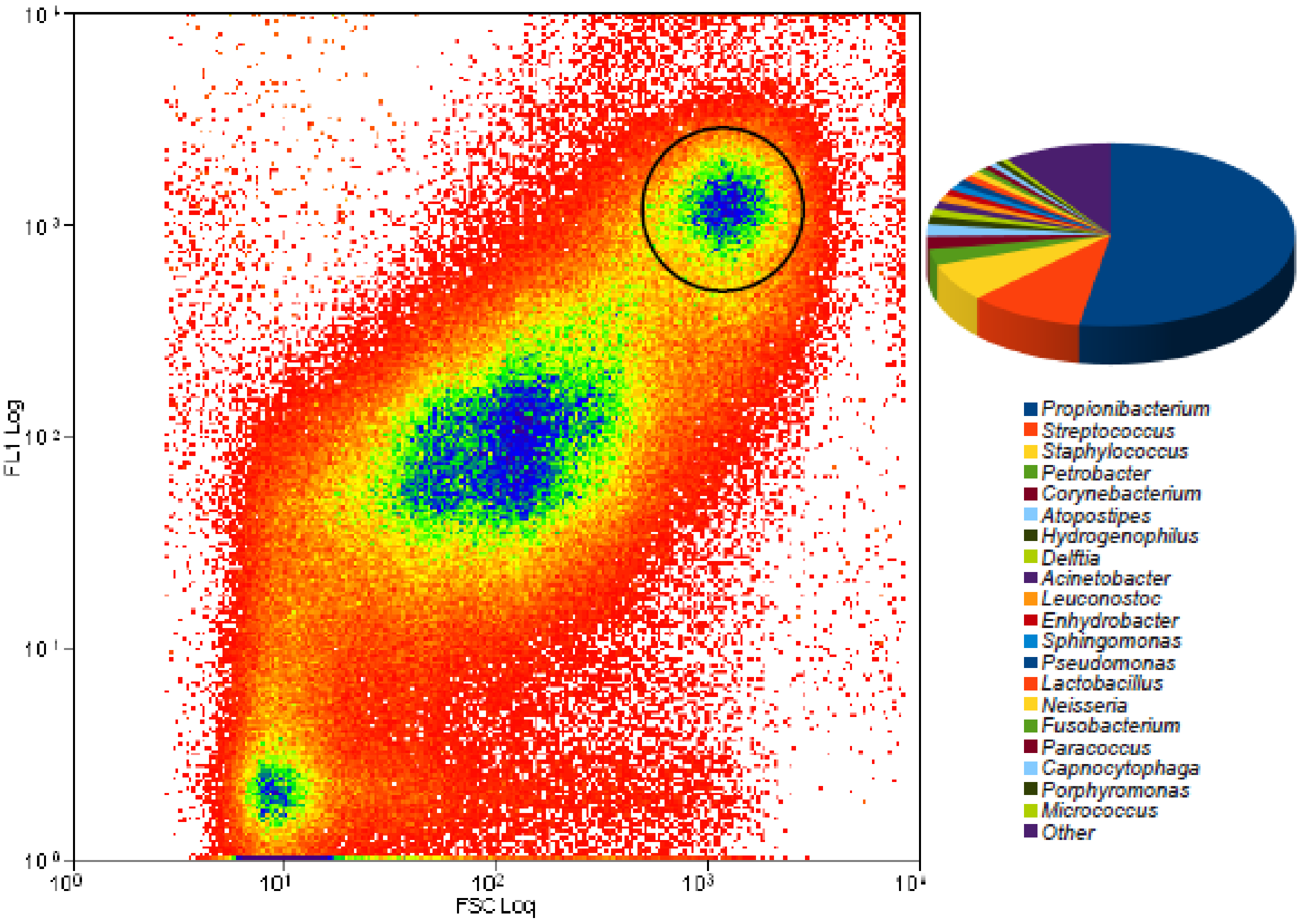

The scatterplot shows microbial cells in a saliva sample according to their size (X axis) and the IgG coating, as indicated by their FITC-fluorescence using anti-human IgG markers (Y axis). The large-size aggregate (indicated with a black circle) was separated by fluorescence-activated cell-sorting and its DNA pyrosequenced after PCR of the 16S rRNA gene, describing its bacterial diversity at the genus taxonomic level. The bacterial populations displaying FITC fluorescence values below 10 correspond to non-opsonized cells.
